# Synthesis and properties of Sr_2_La_2_NiW_2_O_12_, a new *S* = 1 triangular lattice magnet

**DOI:** 10.1107/S2052520624007091

**Published:** 2024-08-30

**Authors:** Anastasiia Smerechuk, Ana Guilherme Buzanich, Bernd Büchner, Sabine Wurmehl, Ryan Morrow

**Affiliations:** a Leibniz Institute for Solid State and Materials Research IFW Dresden Helmholtzstraße 20 Dresden 01069 Germany; b Federal Institute for Materials Research and Testing (BAM) Richard-Willstaetter-Str. 11 Berlin 12489 Germany; Wilfrid Laurier University, Waterloo, Ontario, Canada

**Keywords:** frustrated magnetism, complex oxides, perovskite, X-ray diffraction, triangular magnet

## Abstract

Magnetic materials featuring triangular arrangements of spins are frequently investigated as platforms hosting magnetic frustration. Hexagonal perovskites with ordered vacancies serve as excellent candidates for two-dimensional triangular magnetism due to the considerable separation of the magnetic planes. In this work, the effects of chemical pressure on the ferromagnetic ground state of Ba_2_La_2_NiW_2_O_12_ by substitution of Ba^2+^ with Sr^2+^ to produce Sr_2_La_2_NiW_2_O_12_ are investigated. The two materials are characterized using synchrotron-based XRD, XANES and EXAFS in addition to magnetometry in order to correlate their crystal structures and magnetic properties. Both materials form in space group *R*3, yet as a result of the enhanced bending of key bond angles due to the effects of chemical pressure, the *T*_C_ value of the magnetic Ni^2+^ sublattice is reduced from ∼6 K in Ba_2_La_2_NiW_2_O_12_ to 4 K in Sr_2_La_2_NiW_2_O_12_.

## Abstract 2

Sr_2_La_2_NiW_2_O_12_ is synthesized and compared with Ba_2_La_2_NiW_2_O_12_ as a chemical-pressure analog in order to draw conclusions about the structure–property relationships regarding the triangular magnetism of the Ni sublattice.

## 1. Introduction

Frustrated magnetism, typically defined empirically as the case where a large ratio exists between the Weiss temperature and the onset temperature of long-range magnetic order, has been suggested to arise from a variety of fundamental origins. However, for the realization of a maximally frustrated magnetic system as it is manifested in various theoretically proposed spin liquids (Balents, 2010 ▸; Savary & Balents, 2017 ▸), one nearly universal ingredient is the implementation of geometric spatial arrangement of the spins in the solid which yields a fine balancing of the exchange interaction(s) between them. As highly degenerate energy levels of the ground state are typically associated with such an entanglement, even small perturbations of the crystal structure can result in preferential solutions to alleviate the frustration. As theoretical models can avoid this contingency, many of the theoretical constructs involve simple idyllic patterns as templates for calculations such as the famous kagome or honeycomb lattices.

One of the simplest of such frustrated arrangements, frequently used as an example for introductory purposes, is the antiferromagnetic (AFM) interaction on a triangle, and by extension, tilings in two and three dimensions which are based on triangular arrangements, such as the edge-sharing tetrahedra found in the f.c.c. lattice. While the kagome lattice, also based on triangles, has maintained its status as a frustrated system, the completely tiled 2D triangular plane has received less attention due to the solution to the system which commonly materializes – the 120° AFM structure. However, recent works in the frustrated cobaltate magnet systems suggest the possibility of Kitaev interactions between high-spin *d*^7^ Co^2+^ at low temperatures which may yet again produce interesting results even in 3*d* transition metal triangular magnets (Liu & Khaliullin, 2018 ▸; Kim *et al.*, 2021 ▸, 2023 ▸).

While triangular patterns are very commonly observed in a wide variety of crystalline lattices, simple chemical formulae such as binaries frequently bring neighboring magnetic planes in close proximity, allowing for the out-of-plane terms to significantly impact the properties. Complex oxides, on the other hand, enable for rather exotic and elaborate crystal structures to be designed resulting in well separated 2D planes and more idealistic correspondence to theoretical models. Hexagonal perovskites, one such class of materials, have been studied for many reasons, but recent interest has shown these materials to include excellent candidates for low-dimensional triangular magnets and quantum materials (Nguyen & Cava, 2021 ▸).

One such variant of hexagonal perovskite, first demonstrated in the 1960s (Longo *et al.*, 1965 ▸), has a generalized quadruple perovskite formula (*A*_4_*B*_4_O_12_) convoluted by both cation and vacancy ordering to yield a formula of *A*_4_*BB*′_2_O_12_. The crystal chemistry of these vacancy and cation ordered hexagonal perovskites was elaborated considerably in the 1980s in the group of Kemmler-Sack (Herrmann & Kemmler-Sack, 1980*a* ▸; Rother & Kemmler-Sack, 1980 ▸; Herrmann & Kemmler-Sack, 1981 ▸; Herrmann & Kemmler-Sack, 1980*b* ▸; Kemmler-Sack & Herrmann, 1980 ▸), demonstrating some of the chemical flexibility enjoyed by its more commonly known simple perovskite parents. Apparently abandoned for several decades, these materials have recently resurfaced in the context of a materials platform to study low-dimensional and frustrated magnetism (Evans *et al.*, 2021 ▸).

While many potential members remain yet unexplored, several teams have published results which begin to paint a picture of the interesting accessible properties in these systems. Recent research has focused on inclusion of a single magnetic ion at the *B* site with an oxidation state of 2+, with focus on the magnetism of triangular lattices of Mn^2+^, Co^2+^ and Ni^2+^ (Doi *et al.*, 2017 ▸; Rawl, Lee *et al.*, 2017 ▸; Kojima *et al.*, 2018 ▸; Saito *et al.*, 2019 ▸). Meanwhile, the counterbalancing *A* and *B*′ cations typically are all nonmagnetic to yield a clean 2D magnetic system. The *A* cations utilized thus far have been a combination of alkaline earth and rare earth cations typical of hexagonal perovskites, while the *B*′ cations include *d*^0^ configuration ions such as Re^7+^, W^6+^, Te^6+^ and Nb^5+^. The combinations of charge balance between the *A* and *B*′ sublattices allow for numerous combinations of nonmagnetic scaffolding for each magnetic lattice, in turn allowing for studies with the application of chemical pressure by aliovalent or isovalent cation substitutions (Rawl, Lee *et al.*, 2017 ▸). Furthermore, materials with yet another doubling of the formula and greater dilution of the magnetic layers in these materials produce even greater separation between the layers in related *A*_8_*BB*′_6_O_24_ compounds (Rawl, Ge *et al.*, 2017 ▸, 2019 ▸).

In this work, the topic of investigation concerns the magnetism of Ni^2+^ in *A*_4_*BB*′_2_O_12_ materials. Two such compounds have been reported thus far with considerable difference in properties. Ba_2_La_2_NiTe_2_O_12_ features AFM interactions on the triangular lattice, with two magnetic transitions upon cooling: from paramagnetic to a collinear AFM structure and then to a 120° structure (Saito *et al.*, 2019 ▸). Conversely, Ba_2_La_2_NiW_2_O_12_ exhibits ferromagnetic (FM) interactions and a low-temperature long-range-ordering transition to an FM state (Rawl, Lee *et al.*, 2017 ▸). In order to shed further light on the surprising system, in this work we synthesize additional member Sr_2_La_2_NiW_2_O_12_, effectively applying chemical pressure to Ba_2_La_2_NiW_2_O_12_. Both of these materials are characterized for their magnetic properties and crystal structures using synchrotron X-rays for direct comparison, both to each other as well as to recent neutron powder diffraction results (Yu *et al.*, 2023 ▸).

## 2. Experimental

Polycrystalline samples of Sr_2_La_2_NiW_2_O_12_ and Ba_2_La_2_NiW_2_O_12_ were synthesized in high-density alumina crucibles using the solid-state method. Stoichiometric quantities of La_2_O_3_, SrCO_3_ or BaCO_3_, NiO and WO_3_ were weighed, thoroughly ground in an agate mortar and pestle, and heated in a muffle furnace at 1250°C for a period of 24 h. The rate of heating to the dwell temperature was 100°C h^−1^, while the rate of cooling after dwelling was 50°C h^−1^. Prior to weighing, the La_2_O_3_ reagent had been dried overnight in a muffle furnace at 800°C.

For the preliminary characterization by in-house powder X-ray diffraction (XRD), the samples were finely ground and mounted on to a thin-film sample holder, to be analyzed on a Stoe Stadi diffractometer, in transmission geometry with Co *K*α_1_ radiation, equipped with a Ge monochromator and a DECTRIS MYTHEN 1K detector. Powder XRD data were collected at room temperature (∼293 K) on beamline P02.1 (PETRA III) with an energy of approximately 60 keV and λ = 0.2073 Å (Dippel *et al.*, 2015 ▸). Data were acquired on a Perkin Elmer XRD1621 CN3-EHS (200 µm × 200 µm pixel size, 2048 × 2048 pixel area) area detector and integrated using the *Fit2D* program (Hammersley *et al.*, 1996 ▸). Powder XRD data were analyzed with the Rietveld method using the *GSAS EXPGUI* program (Larson & Von Dreele, 2004 ▸; Toby, 2001 ▸).

X-ray absorption spectroscopy (XAS) measurements containing both X-ray absorption near edge structure (XANES) and extended X-ray absorption fine structure (EXAFS) were performed in fluorescence mode at the Ni *K*-edge (8.333 keV) at the BAMline (Buzanich *et al.*, 2023 ▸), located at BESSY II (Berlin, Germany). The incident energy was tuned by a double-crystal monochromator in a Si(111) arrangement (Δ*E*/*E* = 2 × 10^−4^). A 5 cm-long ionization chamber filled with nitro­gen was used to measure the *I*_0_ signal (before the sample). The beam size was 4 mm (horizontal) × 1 mm (vertical). A four-element silicon drift detector was used to collect the fluorescence signal in backscattered mode, as described by Buzanich *et al.* (2023 ▸). The measurement protocol was: 10 eV steps until 20 eV before the edge, followed by 0.25 eV steps until 20 eV above the edge and 2 eV steps until 200 eV above the edge. From then on equidistant *k*-steps were taken (every 0.06 Å) until 16 Å. The data evaluation and treatment were performed by using the *ATHENA* program from the *DEMETER *(Ravel & Newville, 2005 ▸) package. The EXAFS signal was Fourier transformed, by convoluting a Hanning-type window with the signal in *k*-space between 2 and 14 Å^−1^, with tapering parameter *dk* = 2.

The temperature and field dependence of the direct current magnetization of both samples were measured using a Quantum Design SQUID MPMS. The powder samples were contained in size #4 gel capsules, mounted inside straws, and attached to a standard sample stick for insertion into the device. Temperature dependence of the magnetization was collected in an applied magnetic field of 1000 Oe following both field cooled (FC) and zero field cooled (ZFC) protocols in the temperature range of 2 to 350 K. Field dependent measurements were collected at 2 K, starting from a virginal ZFC state, in the applied field range of ±50 kOe. No corrections to the data for the diamagnetism of the sample holder were necessary given the magnitude of the response.

## 3. Results and discussion

**Figure 1:**
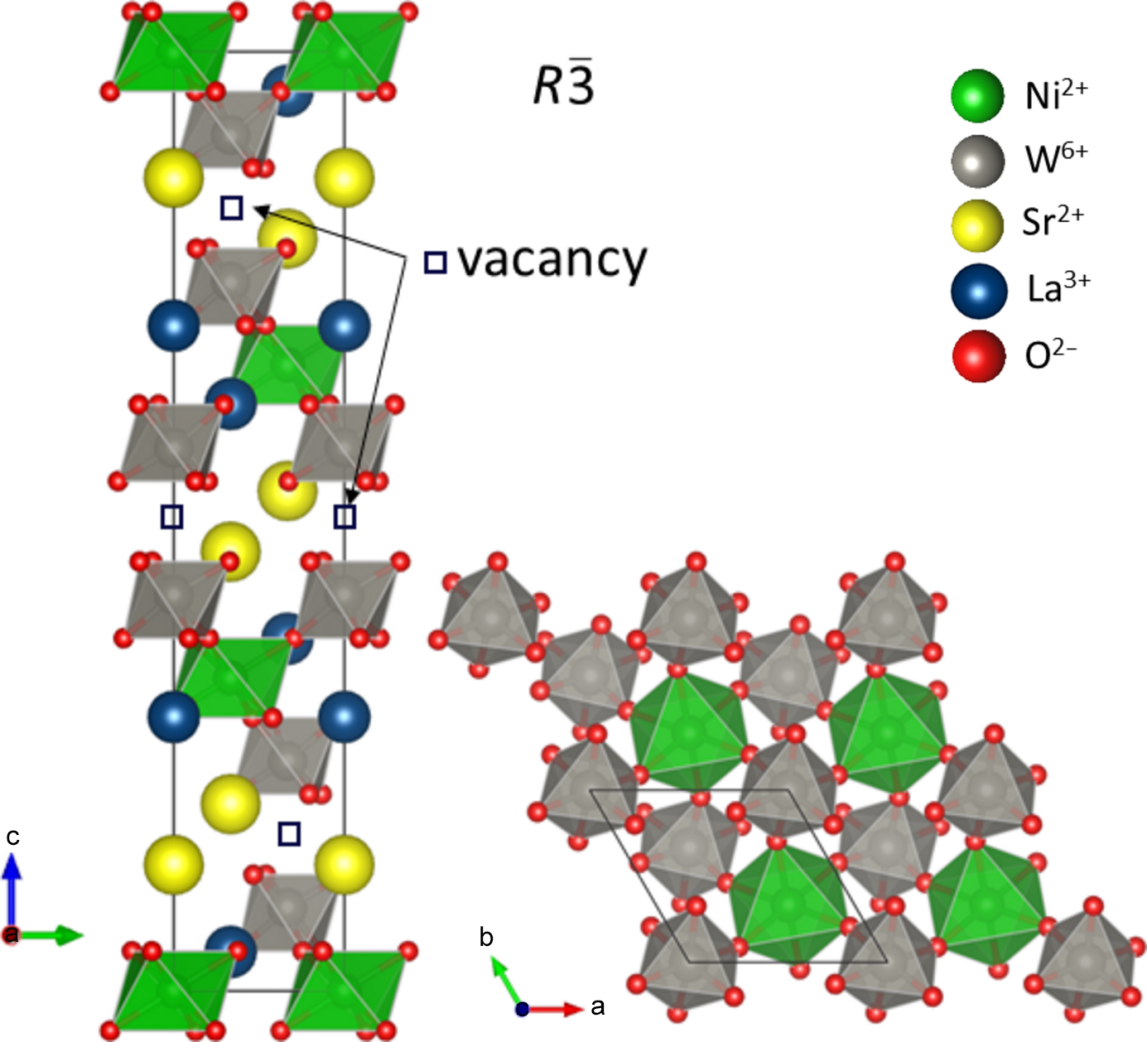
Representation of the crystal structure of Sr_2_La_2_NiW_2_O_12_ viewed along the *a* axis, highlighting the ordered cation vacancies (left), and a cross sectional perovskite-like slab viewed along the *c* axis showing the triangular arrangement of magnetic Ni ions (bottom right). This figure was drawn using the *VESTA* (Momma & Izumi, 2011 ▸) software package.

Both samples crystallize in rhombohedral space group 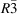 with an overall crystal structure similar to a quadrupled hexagonal perovskite (Nguyen & Cava, 2021 ▸). The primary difference is that the trimer of face-sharing octahedra normally found in such a hexagonal perovskite instead contains an ordered vacancy in the center of this trimer. The location of these sites is highlighted in Fig. 1 ▸. The severed trimers are linked by corner-sharing connections to NiO_6_ octahedra. Much like the more typical hexagonal perovskites, both the face-sharing columns and the linking octahedra are arranged in a triangular layout when viewed down the *c* axis, which has lent hexagonal perovskites opportunities to serve as platforms for low-dimensional and frustrated magnetism. The distance separating the nearest-neighbor Ni ions is equivalent to the *a* unit-cell parameter in each material. However, notably, there is no direct connection via shared ligands between neighboring NiO_6_ octahedra. Therefore, while the severed connectivity between the triangular planes weakens potential out-of-plane exchange interactions, the exchange mechanisms within the plane are also longer ranged and, therefore, of marginal strength. The consideration of a direct exchange between the nickel ions as a potential mechanism is supplemented by superexchange pathways which take into account the neighboring chemical scaffolding. The bonding angle considering a pathway from Ni through W to the nearest Ni is just shy of 90°, meanwhile, there exists the possibility for exchange to proceed via a Ni–O–O–Ni pathway. Interestingly, it appears that the comparison of Ba_2_La_2_NiW_2_O_12_ and nearly isostructural Ba_2_La_2_NiTe_2_O_12_ directly probes these pathways. While Te^6+^ has a full *d* shell, participation in superexchange is precluded, and the remaining mechanism of Ni–O–O–Ni lends an AFM exchange and resultant properties. Meanwhile, Ba_2_La_2_NiW_2_O_12_ has an empty *d* shell in the chemical scaffolding in W^6+^. Apparently, the availability of these orbitals for participation in a super-superexchange mechanism is sufficient to overtake the AFM Ni–O–O–Ni mechanism, which likely persists. The nearly 90° exchange pathway through W yields an FM exchange, much like what is seen in the Goodenough–Kanamori rules (Goodenough, 1955 ▸; Kanamori, 1959 ▸) of simpler directly linked polyhedra. Interestingly, it is highly rational that both mechanisms should be sensitive to any distortions or rotations of the octahedra and resulting oxygen position.

**Figure 2:**
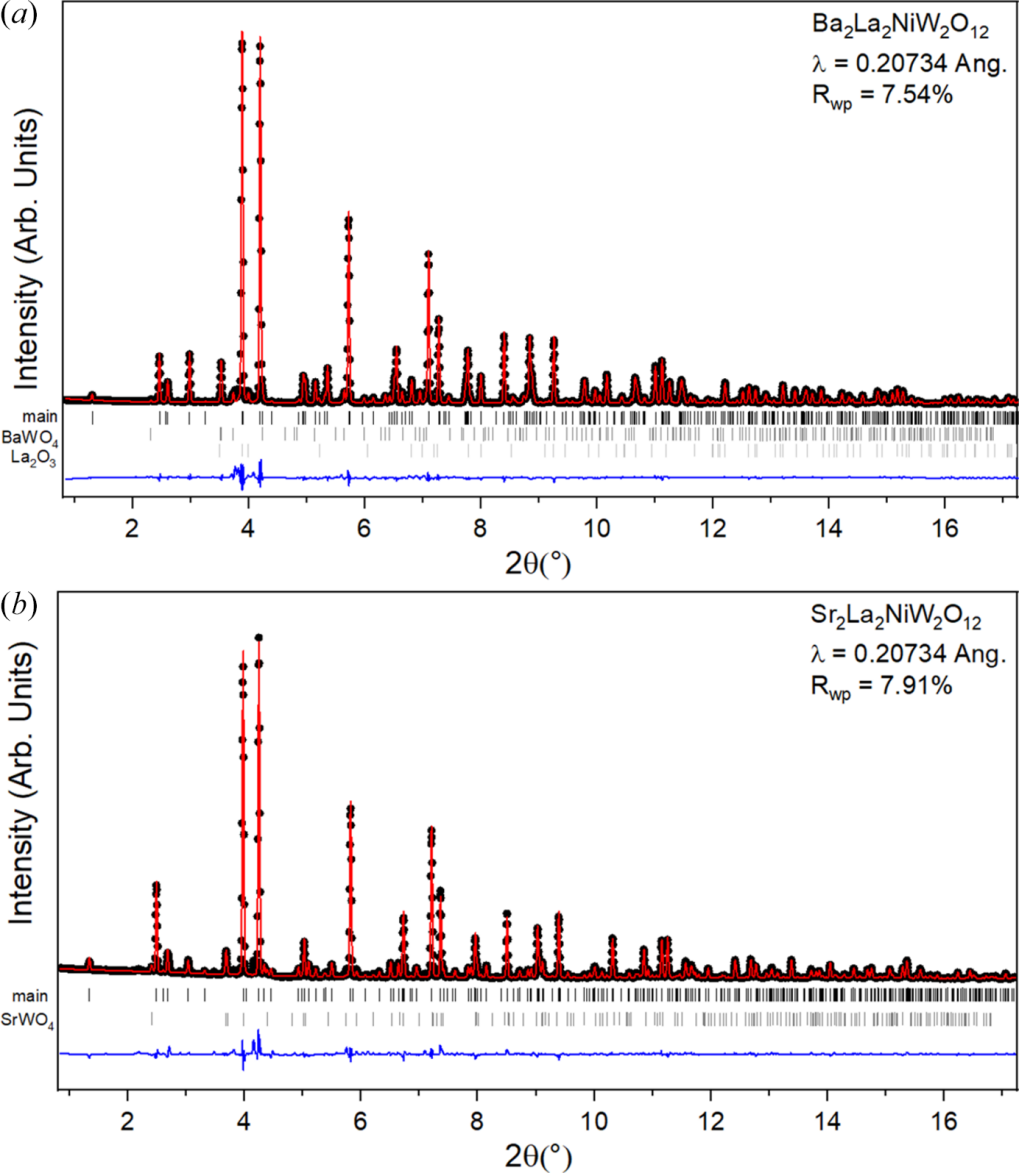
Synchrotron XRD patterns collected at room temperature (∼293 K) of (*a*) Ba_2_La_2_NiW_2_O_12_ and (*b*) Sr_2_La_2_NiW_2_O_12_. The black symbols, red curve and blue curve correspond to the observed data, calculated pattern and difference curve, respectively. Black vertical bars (top) refer to Bragg angles of the main phase while the secondary ones below refer to (*a*) BaWO_4_ and La_2_O_3_ and (*b*) SrWO_4_.

The refinement of the crystal structure models against synchrotron powder XRD data are shown visually in Fig. 2 ▸ and numerically in Table 1 ▸. Two structural models have been proposed in the literature for similar materials with space groups 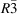 and 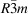 (Saito *et al.*, 2019 ▸), including a recent comparison and assignment of 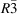 for Sr_2_La_2_NiW_2_O_12_ and Ba_2_La_2_NiW_2_O_12_ on the basis of neutron diffraction (Yu *et al.*, 2023 ▸). The primary difference between the two structural models is that the 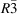 model includes an out-of-phase rotation between neighboring corner-connected octahedra within the perovskite-like slabs as can be seen in Fig. 1 ▸, whereas the 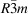 model does not (see supporting information, Fig. S1 ▸). While neutron diffraction is more sensitive to the differences in these structural models as they primarily involve oxygen positions, there is a statistically significant difference in the fitting of the synchrotron XRD patterns favoring the octahedral tilt model associated with 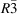, which is consistent with the previous report (Yu *et al.*, 2023 ▸). The refined parameters of both structural models for both materials are given in Table 1 ▸ for direct comparison.

The structural impact of the cation substitution can be clearly seen by comparing the structural models of the two materials. The substitution of smaller Sr^2+^ for Ba^2+^ creates chemical pressure by compressing the material to alleviate potential underbonding of the relatively smaller cation. This can be seen in the unit-cell parameters and unit-cell volume, which are noticeably reduced. Important effects of this compression include the reduced distance between neighboring Ni^2+^ ions and the change in bonding angles implicated in the superexchange mechanisms previously described. In order to verify the accuracy of the bond lengths and angles of importance, it is instructive to compare directly to the neutron diffraction results which can be presumed to more accurately determine the oxygen positions. In Ba_2_La_2_NiW_2_O_12_ and Sr_2_La_2_NiW_2_O_12_, respectively, the Ni—O2 bond lengths are given as 2.064 (4) Å and 2.051 (2) Å, the W—O2 bond lengths as 2.009 (6) Å and 2.004 (2) Å, and ∠Ni—O2—O2 bond angles as 121.50 (5)° and 120.62 (4)° (Yu *et al.*, 2023 ▸). While these values are qualitatively similar to those refined and presented in Table 1 ▸ in the present work, the refinement based on synchrotron X-ray data places the O2 anions closer to W. Presuming a higher precision of the oxygen position refined from neutron data, this results in a slight distortion of the octahedra and bond angles. Meanwhile, the *a* unit-cell parameters of 5.66126 (9) Å and 5.59654 (5) Å and *c* unit-cell parameters of 27.35363 (3) Å and 26.58389 (1) Å given for Ba_2_La_2_NiW_2_O_12_ and Sr_2_La_2_NiW_2_O_12_, respectively (Yu *et al.*, 2023 ▸), compare very closely to the refined values given in Table 1 ▸.

Several impurities exist in the samples which could be accounted for in the refinement process. The Sr_2_La_2_NiW_2_O_12_ sample contains 4.24 (9)% of SrWO_4_ by weight fraction, while the Ba_2_La_2_NiW_2_O_12_ sample contains 5.99 (11)% BaWO_4_ and 0.42 (3)% La_2_O_3_. As the impurities determined were nonmagnetic, they were not considered detrimental to subsequent magnetic characterizations after attempts to purify the samples by revised synthesis were not entirely successful.

**Figure 3:**
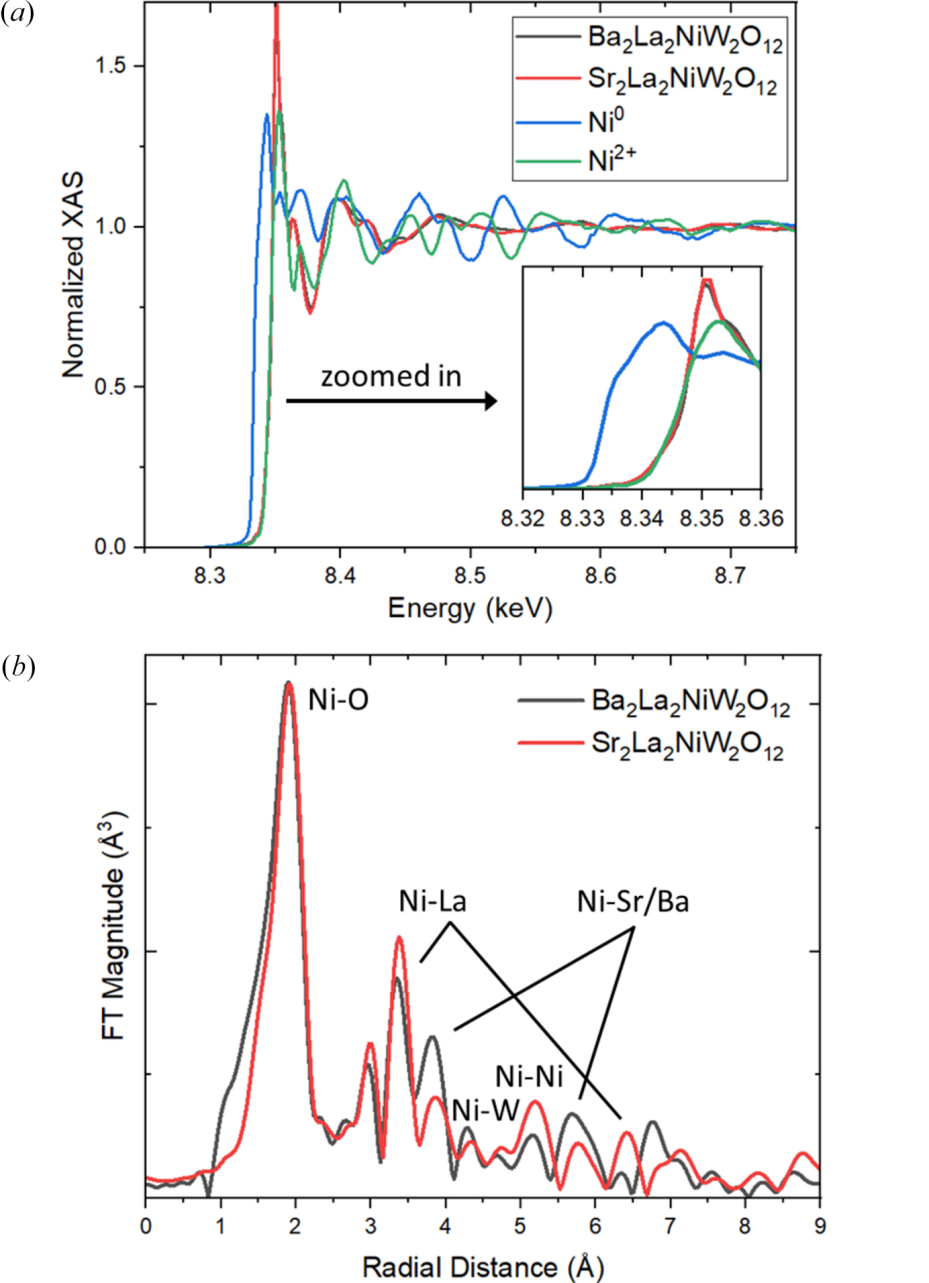
(*a*) Normalized XAS data measured at room temperature near the Ni *K*-edges of Ba_2_La_2_NiW_2_O_12_, Sr_2_La_2_NiW_2_O_12_, standard Ni^0^ foil, and standard Ni^2+^O. The inset displays a close up of the edge position. (*b*) The Fourier transformed EXAFS component, with peaks corresponding to distances from Ni to neighboring pairs, are labeled in the figure.

The assumption that the magnetic Ni ions were in the 2+ oxidation state was tested by XAS measurements using hard X-rays near the Ni *K*-edge (8.333 keV) as shown in Fig. 3 ▸. Standards used in the measurement for comparison purposes were a Ni metal foil and NiO. As shown in the inset of Fig. 3 ▸(*a*), the position of the edge for both samples is directly beneath the signal from the Ni^2+^ standard, signifying a strong agreement with the assumption of the nominal oxidation state. Furthermore, the EXAFS components of the data were able to be analyzed to produce pair correlations corresponding to the local structure radiating from Ni centers. From the basis of the refined averaged crystal structure, tentative assignments of the pair distances to neighboring species could be made. The similarity of the crystal structures is immediately evident in the distribution of the peaks of the two samples. The relatively small change in unit-cell parameters makes little impact on the curves, yet in this data, there is the possibility of checking for cation disorder, namely, that the smaller Sr^2+^ may yield antisite disorder with La^3+^ whereas Ba^2+^ does not seem to. We can observe a similar radial distribution of the peaks between both samples, even at higher radial distances. Noteworthy are the peaks at ∼3.8 Å and ∼5.8 Å, which can arise from Ni–Sr and Ni–Ba scattering paths, respectively. The magnitude in the Ni–Ba case is higher than Ni–Sr, due to stronger scattering from Ba than from Sr. In addition, if there is a distortion prone to happen in the case of Sr, this would produce a lower magnitude in the scattering paths at those distances.

**Figure 4:**
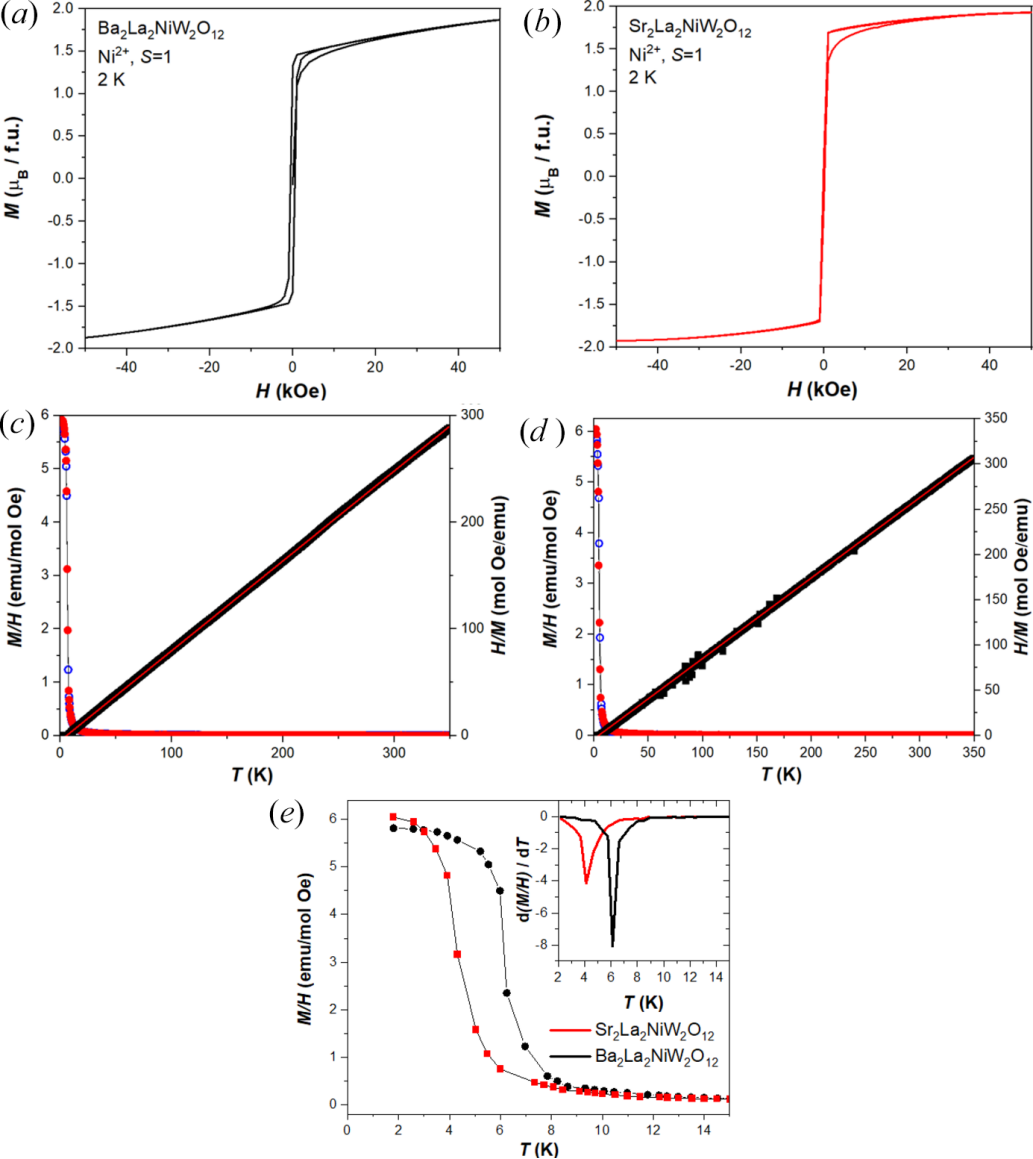
The field dependence of the magnetization of (*a*) Ba_2_La_2_NiW_2_O_12_ and (*b*) Sr_2_La_2_NiW_2_O_12_ at 2 K, the temperature dependence of the magnetization of (*c*) Ba_2_La_2_NiW_2_O_12_ and (*d*) Sr_2_La_2_NiW_2_O_12_ with FC conditions (red) and ZFC conditions (blue) plotted against the left axis and with the inverse of the FC dataset (black) plotted against the right axis with a fitting described in the main text, and (*e*) displays the comparison of the low-temperature FC data of both compounds with the derivative of both plotted in the inset.

Having established these details, we now turn to examine the magnetic behavior of the samples. Both samples have an apparently FM ground state as witnessed in their low-temperature hysteretic field dependence, as shown in Figs. 4 ▸(*a*) and 4 ▸(*b*). Considering the given Ni^2+^ oxidation state, the preponderance of Ni^2+^O_6_ octahedra in the literature establish a high-spin *d*^8^*S* = 1 electronic configuration as the most reasonable expectation. Indeed, the saturation magnetization of both compounds is very nearly the ideal 2 μ_B_ per Ni^2+^ with 1.87 μ_B_ and 1.93 μ_B_ in Ba_2_La_2_NiW_2_O_12_ and Sr_2_La_2_NiW_2_O_12_, respectively. In light of the presence of approximately 6% and 4% of nonmagnetic impurities in the two samples, a correction accounting for the precise moles of magnetic species would bring these two numbers even closer together and even closer to the nominal value of 2 μ_B_. Given the proximity of these values to the nominal value, interpretations of the ground state as anything more complex than a trivial collinear ferromagnet can be considered unreasonable. Both materials are soft ferromagnets, with low coercive fields, and with Sr_2_La_2_NiW_2_O_12_ being softer than Ba_2_La_2_NiW_2_O_12_.

The temperature dependence of the magnetization reveals FM transitions in both materials at low temperatures. The temperatures of these transitions can be best assigned on the basis of the derivative of the magnetic susceptibility with respect to temperature, as shown in the inset of Fig. 4 ▸(*e*). *T*_C_ lies just above 6 K and 4 K in Ba_2_La_2_NiW_2_O_12_ and Sr_2_La_2_NiW_2_O_12_, respectively, within resolution of the temperature spacings measured. These values agree are consistent with those previously reported (Yu *et al.*, 2023 ▸) on the basis of several methods. The higher temperature portion of both data sets adheres to a well behaved Curie–Weiss law, as shown in Figs. 4 ▸(*c*) and 4 ▸(*d*). The effective moments of both samples are similar with 3.08 μ_B_ and 3.00 μ_B_ for Ba_2_La_2_NiW_2_O_12_ and Sr_2_La_2_NiW_2_O_12_, respectively. These values can be compared to a spin only effective moment of 2.83 μ_B_, with a positive orbital contribution typical of a *d*^8^ configuration. The Weiss temperatures were +5.83 K and +5.40 K for Ba_2_La_2_NiW_2_O_12_ and Sr_2_La_2_NiW_2_O_12_, respectively, highlighting the presence of FM interactions. The corresponding literature values reported for Ba_2_La_2_NiW_2_O_12_ by Rawl, Lee *et al.* (2017 ▸) were 3.19 μ_B_ for the effective moment, +25.5 K for the Weiss temperature, and with a *T*_C_ value of 6.2 K while those reported by Yu *et al.* (2017 ▸) for Ba_2_La_2_NiW_2_O_12_ and Sr_2_La_2_NiW_2_O_12_, respectively, are effective moments of 3.17 μ_B_ and 3.13 μ_B_, Weiss temperatures of 7.4 K and 8.4 K, and *T*_C_ values of 4.3 and 4.8 K.

The results, combined, indicate that the substitution of Sr for Ba effectively applies chemical pressure to the system, reducing the unit-cell parameters and twisting bond angles as a result. The effect of these structural changes on the magnetism are to lower the *T*_C_ by a considerable degree, on the relative low-temperature scale. While naively, one may expect that the chemical pressure serves to bring the Ni ions closer together, which it does, and to enhance the exchange interactions as a result. This would be true if direct exchange were the responsible mechanism. However, it is clear from the change in the sign of the exchange between Ba_2_La_2_NiW_2_O_12_ and Ba_2_La_2_NiTe_2_O_12_, that direct exchange is not responsible. Therefore, the bending of the bond angles, and its implications for the longer-range indirect exchange mechanisms must be the cause. The reduced *T*_C_ can then be considered as weakening of the FM exchange as a result of the less ideal bond angles, it can be considered as a strengthening of the competing AFM Ni–O–O–Ni mechanism, which manifests in the ground state of Ba_2_La_2_NiTe_2_O_12_, or as a combination of the two effects.

## Supplementary Material

Figure S1. DOI: 10.1107/S2052520624007091/dv5014sup2.pdf

Crystal structure: contains datablock(s) SR2LA2NIW2O12_SYNCH4_publ, SR2LA2NIW2O12_SYNCH4_overall, SR2LA2NIW2O12_SYNCH4_phase_1, SR2LA2NIW2O12_SYNCH4_phase_2, SR2LA2NIW2O12_SYNCH4_phase_3, SR2LA2NIW2O12_SYNCH4_phase_4, SR2LA2NIW2O12_SYNCH4_phase_5, SR2LA2NIW2O12_SYNCH4_p_05. DOI: 10.1107/S2052520624007091/dv5014sup2.cif

## Figures and Tables

**Table 1:** Crystallographic data from synchrotron XRD for Ba_2_La_2_NiW_2_O_12_ and Sr_2_La_2_NiW_2_O_12_ in both space groups considered in the text In both space groups, coordinates Ba/Sr *x*, Ba/Sr *y*, La *x*, La *y*, Ni *x*, Ni *y*, Ni *z*, W *x* and W *y* are equal to 0.

	Ba_2_La_2_NiW_2_O_12_	Ba_2_La_2_NiW_2_O_12_	Sr_2_La_2_NiW_2_O_12_	Sr_2_La_2_NiW_2_O_12_
Space group	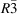	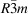	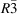	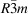
*a* (Å)	5.66176 (8)	5.66167 (8)	5.59271 (8)	5.59271 (8)
*c* (Å)	27.3620 (6)	27.3623 (6)	26.5639 (6)	26.5638 (6)
*V* (Å^3^)	759.59 (3)	759.58 (3)	719.56 (3)	719.56 (3)
*R*_wp_ (%)	7.54	7.81	7.91	8.06
χ^2^	158.9	170.2	147.1	152.2
Ba/Sr *z*	0.13276 (13)	0.13285 (15)	0.13430 (16)	0.13433 (16)
La *z*	0.29296 (17)	0.29276 (17)	0.29173 (13)	0.29158 (13)
W *z*	0.41860 (14)	0.41848 (14)	0.42188 (10)	0.42176 (10)
O1 *x*	0.483 (6)	0.4951 (14)	0.460 (9)	0.4966 (15)
O1 *y*	0.498 (6)	0.5049 (14)	0.467 (9)	0.5034 (15)
O1 *z*	0.1175 (4)	0.1174 (4)	0.1238 (4)	0.1232 (4)
O2 *x*	0.432 (3)	0.4841 (12)	0.422 (3)	0.4811 (15)
O2 *y*	0.462 (3)	0.5160 (12)	0.462 (3)	0.5189 (15)
O2 *z*	0.2944 (5)	0.2947 (5)	0.2910 (5)	0.2914 (4)
∠Ni—O2—W (°)	160.6 (8)	169.8 (7)	159.4 (8)	171.0 (8)
∠Ni—W—Ni (°)	89.65 (6)	89.70 (7)	88.85 (5)	88.91 (6)
∠Ni—O2—O2 (°)	122.9 (8)	122.9 (8)	147.9 (5)	137.4 (5)
Ni—O2 (Å)	2.10 (3)	2.10 (3)	2.15 (3)	2.11 (3)
W—O1 (Å)	1.79 (5)	1.815 (11)	1.85 (6)	1.83 (6)
W—O2 (Å)	1.98 (2)	1.950 (12)	1.91 (2)	1.89 (3)

## Data Availability

The authors confirm that the data supporting the findings of this study are available within the article and its supplementary materials.
