# Supplementary material for: Synthesis and properties of Sr2La2NiW2O12, a new S = 1 triangular lattice magnet
Source: Acta Crystallogr B Struct Sci Cryst Eng Mater. 2024 Aug 30;80(Pt 5):467–73. doi: 10.1107/S2052520624007091 (PMC11457098; doi:10.1107/S2052520624007091)
Supplement: Supplementary file 1 [file b-80-00467-sup2.pdf]

STRUCTURAL SCIENCE
CRYSTAL ENGINEERING
MATERIALS

**Volume 80 (2024)**

**Supporting information for article:**

# Synthesis and Properties of Sr2La2NiW2O12, a new *S* = 1 triangular lattice magnet

**Anastasiia Smerechuk, Ana Guilherme Buzanich, Bernd Büchner, Sabine Wurmehl and Ryan Morrow**

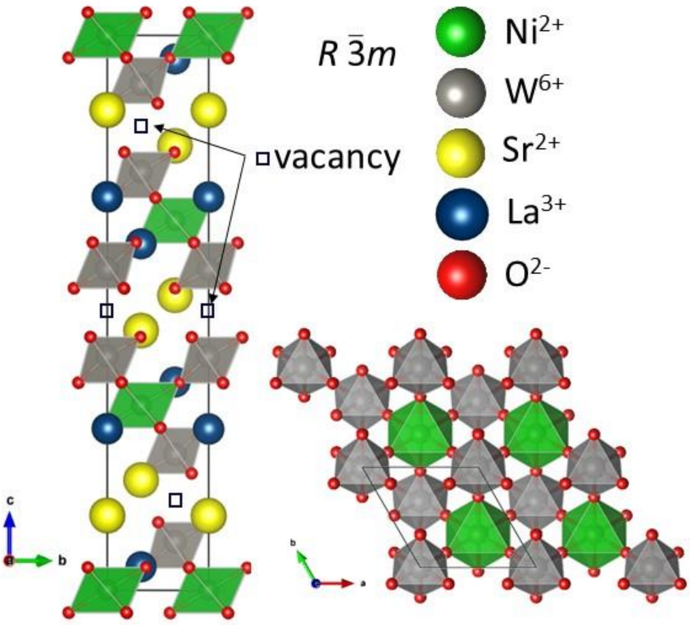


**Figure S1.** Representation of the crystal structure of $Sr_2La_2NiW_2O_{12}$ viewed along the *a* axis, highlighting the ordered cation vacancies (left), and a cross sectional perovskite-like slab viewed along the *c* axis showing the triangular arrangement of magnetic Ni ions (bottom right). Figures were drawn using the VESTA software package. This figure differs from main Figure 1 in that the space group is $R\bar{3}m$ and the octahedral tilting found in the $R\bar{3}$ space group are absent.
